# Episcopic 3D Imaging Methods: Tools for Researching Gene Function

**DOI:** 10.2174/138920208784533601

**Published:** 2008-06

**Authors:** Wolfgang J Weninger, Stefan H Geyer

**Affiliations:** IMG, Centre for Anatomy and Cell Biology, Medical University of Vienna, Waehringer Str. 13, A-1090 Vienna, Austria

**Keywords:** 3D modelling, episcopic microscopy, imaging, embryo, development, gene expression.

## Abstract

This work aims at describing episcopic 3D imaging methods and at discussing how these methods can contribute to researching the genetic mechanisms driving embryogenesis and tissue remodelling, and the genesis of pathologies. Several episcopic 3D imaging methods exist. The most advanced are capable of generating high-resolution volume data (voxel sizes from 0.5x0.5x1 µm upwards) of small to large embryos of model organisms and tissue samples. Beside anatomy and tissue architecture, gene expression and gene product patterns can be three dimensionally analyzed in their precise anatomical and histological context with the aid of whole mount *in situ* hybridization or whole mount immunohistochemical staining techniques. Episcopic 3D imaging techniques were and are employed for analyzing the precise morphological phenotype of experimentally malformed, randomly produced, or genetically engineered embryos of biomedical model organisms. It has been shown that episcopic 3D imaging also fits for describing the spatial distribution of genes and gene products during embryogenesis, and that it can be used for analyzing tissue samples of adult model animals and humans. The latter offers the possibility to use episcopic 3D imaging techniques for researching the causality and treatment of pathologies or for staging cancer. Such applications, however, are not yet routine and currently only preliminary results are available. We conclude that, although episcopic 3D imaging is in its very beginnings, it represents an upcoming methodology, which in short terms will become an indispensable tool for researching the genetic regulation of embryo development as well as the genesis of malformations and diseases.

## BACKGROUND

Challenging hereditary diseases and pathologies requires profound knowledge of the genetic and epigenetic pathways regulating their genesis. Therefore modern biomedicine developed a number of different approaches for researching the role and function of genes and gene products in normal and diseased embryos, organs, and tissues. The following study shall provide a very brief overview of techniques capable of researching gene function. It will then focus on imaging methods, which can quickly generate correctly registered digital image stacks, captured from the block surfaces of sequentially cut, histological processed, and embedded specimens (three dimensional (3D) episcopic imaging methods). These methods will be described in more detail and their value for researching gene function will be discussed.

## ANALYZING GENE FUNCTION – A BRIEF OVERVIEW

Basically three main approaches exist for researching the activity and function of genes and gene products.

### 1 Gene Profiling 

Tissue samples of normal or diseased model organisms or humans can be analyzed with the aid of microarray techniques [1-6]. Microarray studies provide information about the activity of thousands of genes in very short times. They reveal the patterns of gene activity in individual tissues and provide clues about gene function and their association with regulatory or signalling pathways. However, it is difficult to compare multiple micro-array experiments and array-derived data is only of limited value in defining gene function, because it frequently lacks spatial and/or temporal specificity. Thus microarray techniques are highly useful for describing and even quantifying the activity of multiple genes in tissue samples, but in order to properly understand gene function, gene-expression patterns need to be placed in the proper 3D context of tissue architecture. 

### 2 Phenotyping Mutants of Model Organisms 

Model organisms with randomly introduced or targeted gene disruptions are produced and their phenotype is comprehensively examined. This study will not discuss the methods, which are used for analyzing the cognitive or receptive function or the behaviour of animals. It will also not discuss methods designed for analyzing the morphological phenotype of adult animals or humans (e.g. computed tomography, magnetic resonance imaging, ultrasound, etc.). But it will discuss some methods capable of providing morphological descriptions of tissue samples and embryos of model organisms.

### 3 *In Situ* Gene Expression Analysis

Expression patterns of genes and gene products are analyzed in their precise anatomical and histological context. For conducting such analysis methods were developed, which permit, analysis of gene or gene product patterns in the context of tissue architecture, tissue samples, or tissues and organs of model organisms and their embryos respectively.

With the exception of anatomical dissection, traditional techniques for analyzing microscopic and macroscopic anatomy and gene expression patterns, are two-dimensional (2D) (electron-microscopic, histological, and macroscopic sections, etc.). But tissues and organs are three-dimensional (3D) and gene products are distributed and act three dimensionally. Thus the last decades saw the development of a vast number of methods for creating 3D information of cell, tissue, and organ morphology, 3D information of gene expression and gene product patterns, or both. Examples for techniques capable of analyzing small specimens, such as tissue samples or embryos are: *in vivo* microscopy [7-10], micro-computed tomography (µCT) [11-13], micro-magnetic resonance imaging (µMRI) [9, 14-18], ultrasound biomicroscopy (UBM) [19-21], optical projection tomography (OPT) [22, 23], confocal microscopy [24-28], atomic force microscopy [29-31], 3D electron tomography [32, 33], histological or macroscopic section based 3D reconstruction methods [34-38], and 3D episcopic imaging methods (see below).

This paper does not compare all the different methods for volume data generation and gene expression analysis. It will solely focus on the description, analysis and discussion of methods that permit volume data generation on the basis of episcopic images of physically sectioned specimens.

## EPISCOPIC 3D IMAGING METHODS – DEFINITION

Under the term “episcopic 3D imaging methods” we summarize all 3D imaging techniques that create volume data by capturing images of subsequent surfaces of blocks, containing histological processed and embedded specimens, during their physical sectioning on microtomes. This includes methods such as “fast 3D serial reconstruction” [39], “Epi-3D” [40], “episcopic fluorescence image capturing” [41], “surface imaging microscopy” [42, 43], “high resolution episcopic microscopy (HREM)” [44], and serial block-face scanning electron microscopy (SBF-SEM) [45]. The term “surface imaging microscopy” was used twice. We will refer to the earlier method as SIM1 [42] and the later as SIM2 [43]. We will not go into detail with SBF-SEM, which is capable of generating stacks of precisely aligned electron microscopy images and permits highly detailed 3D analysis of cell organelles, axons, and synapses. Although a very sophisticated method, it can neither be used for describing the morphological phenotype of mutants, nor for examining gene expression patterns in the morphological context.

## EPISCOPIC 3D IMAGING METHODS – WORK FLOW

**Fig. (1):**
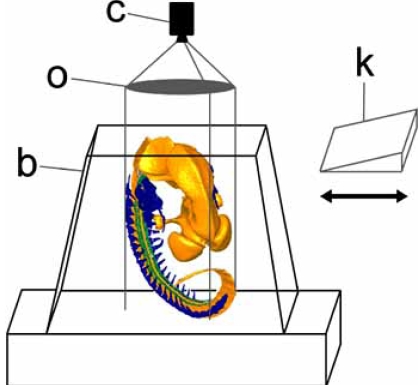
Set up used for episcopic 3D data generation. A camera (c), sitting on an optic (o) is aligned perpendicular to the surface of a block of embedding medium (b) containing a specimen. Note that the field of view (marked by grey lines) should be as narrow as possible. Its position cannot be changed during sectioning. k, knife.

**Fig. (2):**
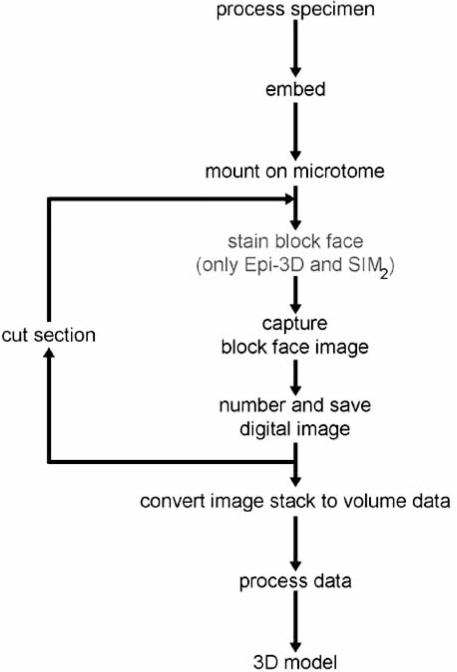
Flow chart of the episcopic 3D data generation protocol. Note that this flow chart provides only the main steps. Depending on the method, additional steps might be necessary for analysing gene expression patterns.

All episcopic 3D imaging techniques utilize sacrificed embryos and tissue samples. The specimens are fixed and sometimes pre-processed and whole mount stained for enhancing contrasts. Then they are embedded in histological embedding media and mounted on a microtome. Digital images of the tissues on the surfaces of the blocks of embedding medium containing the specimens are captured with a camera sitting on a magnifying optic. The optical pathway of the optic is aligned precisely perpendicular to the block surface (Fig. **1**). Depending on the technique the tissues on the block surface are either identified by their intrinsic contrast or by administering dyes to the block surface. After capturing an image of the block face, a small slice of the block is removed using either the microtome blade or micro-mills. Routinely this slice is thrown away, although some methods permit preservation of histological sections. Now, a digital image of the freshly cut - if necessary also freshly stained - block surface is captured and the next slice of embedding medium is removed. This procedure is repeated until the region of interest is sectioned and a stack of aligned digital images, showing subsequent block faces with tissue of the specimens is produced (Fig. **2**). In some methods, data generation is fully automated. This generates homogeneous data sets, and saves time and man power.

**Fig. (3):**
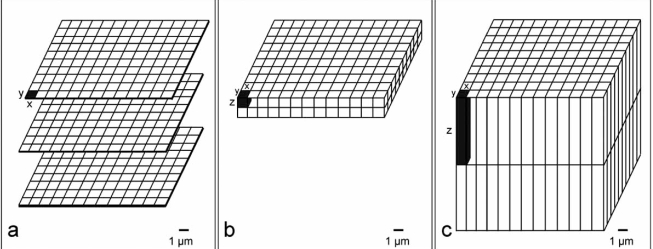
From pixel to voxel. **a.** Schematic drawing of three subsequent two-dimensional (2D) images. Each image consists of 10 x 13 pixels. **b.** Volume data created from images of 1 µm thick sections. The voxels are cubic, because the distance between the images is the same as the length of the x and y coordinate of the pixels of the 2D images. **c.** Volume data created from images of 7 µm thick sections. While the 2D resolution (resolution of the 2D images) is relatively high, the 3D resolution (resolution of the volume data set) is low.

After data generation, the stack of aligned episcopic images is converted into a volume data set. The dimension of one voxel is defined by the resolution of the captured block face image (its Pixel size) and the thickness of the slice of embedding medium removed (Fig. **3**). Virtual volume or surface rendered 3D models can be created and visualized with state of the art 3D visualization software packages (e.g., Amira (Visage Imaging), Volocity (Improvision), SURFdriver (www.surfdriver.com), Osirix (www.osirix-viewer.com)).

## EPISCOPIC 3D IMAGING METHODS – TECHNICAL DETAILS

The following chapter will shortly describe each method and discusses potential applications. The methods are ranked according to their year of publication. Table **1** highlights the characteristics of the different episcopic imaging methods.

“Fast 3D serial reconstruction” [39] is designed and was used for obtaining information on the density of trabeculae in vertebrae [39, 46-48]. The bones become bleached and embedded in black resin, which results in excellent contrasts. Volume data and 3D models of osseous structures can be generated quickly and in high quality. However, the method is restricted to the analysis of the morphology of uniform structures. Due to the improvement of computed tomography, osseous structures can be visualized even faster in similar resolution with modern computer tomography apparatuses, wherefore, as far as we know, the original method is not longer in use. 

“Epi-3D” [40] is designed and was applied for analyzing embryo morphology and the topology of neonate and adult organs respectively [49-52]. After fixation, the specimens become dehydrated and simultaneously pre-stained with lead acetate, before they are embedded in embedding media on wax basis. The histological blocks are sectioned on a conventional microtome. For contrasting the tissues on the block surface, each freshly cut block surface is stained for 1 second with lead acetate. This method permits simple analysis of morphology, but does not offer the possibility for analyzing gene expression patterns. 

“EFIC” [41] is designed and was applied for analyzing the morphology of the organ systems of normal and malformed embryos and for analyzing the topology of diseased tissues [53, 54]. The specimens are embedded in a reddish stained embedding medium on wax basis. Monochrome light of 460 nm wavelength is applied to the block surface in order to excite autofluorence of the tissues. No block surface staining is required for achieving tissue contrast. Therefore the data generation process can be fully automated. In principle the system offers the possibility to visualise the expression patterns of transgenes and gene products, if specimens are whole mount stained with LacZ or NBT/BCIP prior to embedding (http:www.univie.ac.at/efic). The bluish stain extincts tissue autofluorescence, wherefore dyed tissues appear black in the digital images. This “negative contrast” however, hampers decent gene expression analysis. If specifically stained tissues are located next to cavities, lumina, or surfaces, there is no possibility to distinguish between the true border of the cavity, lumen, or surface and the beginning of the stained tissue.

“SIM_1_” [42] is designed and was applied for analysing embryos and tissue samples [55]. It was the first episcopic method that became commercialized. The specimens are embedded in a black embedding medium on resin basis and tissue autofluorescence is used for obtaining tissue information. In principle, SIM_1_ is capable of visualizing multiple gene expression or gene product patterns in whole mount labelled specimens. However, it is bound to use fluorescent antibodies for detecting specifically labelled gene products. Unfortunately reliable whole mount fluorescence staining methods do not exist for many biomedical relevant model organisms. Furthermore the opacity of the embedding medium hampers positioning of the specimens and the definition of the correct field of view to become visualized (see below).

“HREM” [44] is designed and was applied for analyzing embryos and tissue samples [56]. Specimens become immersed and embedded in resin dyed with eosin. The eosin causes an unspecific staining of the tissues. The intrinsic fluorescence of the resin covers shining through effects. The digital images of the block surfaces nearly resemble the quality of digital images of unspecifically stained histological sections (http://www.meduniwien.ac.at/3D-Rekonstr/HREM/). If whole mount LacZ or NBT/BCIP stained specimens are embedded, HREM is capable of visualizing specific gene expression or gene product patterns in the context of morphology and tissue architecture. In principle analysis of multiple gene expression patterns in one specimen are feasible. But they are not possible with the current technology. 

“SIM_2_” [43] is designed and was used for analyzing heart morphology. It uses waxes or resins as embedding mediums and an ultramiller for removing embedding medium from the blocks. The block surfaces are etched and then the freed tissues on the block face are stained for approximately 20 seconds. The staining solutions are similar to traditional histochemical stains, but are optimized for quick staining. SIM_2_ is the youngest of the described methods (2007). Therefore the whole range of potential applications has not yet been explored. Basically it fits for analyzing the tissue architecture of all organ systems and, in principle, it has the potential for analyzing gene expression patterns.

## EPISCOPIC 3D IMAGING METHODS – RESOLUTION OF VOLUME DATA

Most episcopic 3D imaging methods permit data generation of small to large specimens in high to low resolution. But, as in traditional microscopy, increasing the resolution means narrowing the field of view and thus the volume that can be analysed. This results in the simple relation; the larger the specimen to be visualized, the lower the resolution of the volume data. Two ways exist for overcoming this problem. One is to increase the resolution of the scanner or digital camera used for image capturing. However, this is limited by the numerical aperture of the optics, which the light coming from the specimen has to pass to reach the chip of the scanner or camera. Increasing the target size of the scanner without decreasing the numerical aperture of the optics will result in larger digital images without additional information. The second approach to visualize large specimens in high resolution is to raster the block surface with a camera sitting on an optic with low numerical aperture. Multiple, overlapping high-resolution images of one block surface can be captured and digitally stitched together to form a single high resolution block face image. Although true high-resolution images of large specimens can be generated in this way, capturing multiple images from the same block surface is hampered by several problems. The most serious are: 1.) the block surface heats up, which results in artefacts or even breaking of the blocks, and 2.) complicated and expensive data generation apparatuses are required.

The resolution of volume data (voxel size) depends on both, the resolution (Pixel size) of the digital block surface images and on the thickness of the slices of embedding medium removed between capturing two subsequent images (Fig. **3**). The 3D resolution usable for 3D analysis is defined by the largest of these two factors. As an example, images stacks derived from images with Pixel sizes of 0.5x0.5 µm, which were captured in 10 µm distances (voxel size 0.5x0.5x10 µm) are insufficient for analysing single cells, or the topology of capillaries and small embryonic blood vessels. Capillaries (~5 µm diameter) might run for long distances, or might even branch “between” the images of two subsequent 10 µm sections. Such topological information is entirely lost. This is one of the main problems of physical section based 3D reconstruction methods [35-37, 57]. They have to use relatively thick sections in order to reduce the number of sections. By this the time expense for section processing and manual image capturing is kept feasible. Episcopic imaging methods do not have this problem. Capturing of digital images can be performed automatically during the sectioning process and takes only a few seconds. Therefore the time expense for the researcher is independent from the number of digital images captured. Episcopic 3D imaging methods typically generate image series of 500 – 2,000 single images, while producing sections of 1-3 µm thickness within a few hours operating time.

The dimension of one voxel (voxel size) of a data volume provides information about its numeric resolution. However, interpolation errors during data generation and data processing, and other artefacts must be taken into account. Therefore information resting in only one Voxel is unreliable under routine conditions. Furthermore, two cells (or structures) can only be discriminated, if at least one voxel, belonging to neither of them is located in between them. Therefore methods generating data volumes with voxels sizes of, for example, 10x10x10 µm, are numerically cellular, although they effectively are far from being capable of analyzing cells or even cell distributions. For analyzing the shape or for detecting the nature of a cell (or structure) the cell has to cover many voxels in sufficient contrast. Episcopic 3D image methods on average produce volume data of 2x2x2 µm voxel size. Such data are not capable for analyzing cell organelles, but they are of a sufficient resolution to permit rough estimations of cell morphology, cell counts, and analysis of cell distributions.

## EPISCOPIC 3D IMAGING METHODS – ADVANTAGES 

The attractiveness of episcopic 3D imaging methods results from several advantages. The most important are:

### 1 Fast Data Generation

Most episcopic 3D imaging methods are capable of generating digital images of (in most cases) near to histological quality by capturing digital images directly from the surface of the embedding block during sectioning. In contrast to traditional histology and histological section based 3D reconstruction methods [34, 36, 37], no tedious section mounting, section realignment, section processing, and section image capturing is required. Volume data, e.g. consisting of 2,000 single images are available for analysis a few hours after starting the sectioning process. Since data generation can be automated, man-power is only necessary for setting up the system, starting sectioning, as well as subsequent data archiving, data visualisation, and data analysis.

### 2 High Data Quality

Episcopic 3D imaging methods capture images directly from the block surface. If the optic is not moved (Fig. **1**) and decent microtomes are used, the images of the resulting image stack are accurately aligned. Furthermore, similar to CT or MRI data, episcopically captured images do not show distortions introduced by sectioning, section mounting, or section processing. However, like with all post-mortem imaging techniques (including µCT, µMRI, etc.), artefacts introduced by specimen processing (fixation, embedding, etc.) do occur.

### 3 High Resolution

As discussed in the preceding chapter episcopically generated volume data are of high 3D resolution (Fig. **3**). This resolution is unmatched by other 3D methods, except for techniques optimized for analyzing cellular details, such as confocal microscopy, electron microscopy etc.. But data generated with such techniques do not permit spatial analysis of cells in the 3D context of whole embryos or tissue samples.

### 4 Cheap Set Up

Currently no apparatus permitting episcopic volume data generation is commercially available. The single components however, are relatively cheap. They are standard equipment of most biomedical laboratories, and can be easily put together to function as an episcopic data generation apparatus. Nevertheless specialist knowledge is required for data interpretation and trouble shooting.

## EPISCOPIC 3D IMAGING METHODS – TECHNICAL CHALLENGES

Episcopic imaging has three big and several small technical challenges. Here, we will only discuss the major ones.

### 1 Correct Positioning of the Specimens

Once automated data generation has started, the position of the optics in relation to the block must not be changed. Changes inevitably result in misaligned digital images and thus incorrect volume data. Therefore, prior to sectioning, the volume to visualize must be exactly positioned in respect to the optical pathway (Fig. **1**).

### 2 Shining Through Effect

Episcopic 3D imaging methods capture images directly from the surface of blocks of embedding material, which contain the specimen. Only tissue information from the very block face shall be visible in the digital images. However, tissues, which rest deep inside the block do “shine through” to and become visible at the block face. This diminishes contrasts and even obscures details in the block face images.

Epi-3D and SIM_2_ try to overcome the shining through problem by contrasting only the tissues on each freshly cut block surface. However, this complicates automation of block face staining and thus automation of data generation. Unavoidable minimal differences in block face staining time result in different staining intensities between subsequent section images. Ultimately this hampers digital data processing and subsequent 3D modelling. Good section homogeneity and automation can be achieved with all episcopic imaging methods, which do not require block face staining (fast 3D serial reconstruction technique, SIM_1_, EFIC, and HREM). These techniques use dyed embedding materials for eliminating, or at least reducing the shining through effect.

### 3 Block Heating

The illumination of the block surface must be sufficiently bright to achieve tissue contrast by light reflection or by exciting tissues to emit fluorescence signals. The required high intensities of light can cause heating of the block face and subsequently heating of the entire block. This heating may result in expansion of the block, melting of the embedding medium, and even breaking of the block. Automation of data generation, shutter systems, and special compositions of the embedding medium help to reduce the negative effects caused by heating.

## EPISCOPIC 3D IMAGING METHODS – ANALYZING TISSUE ARCHITECTURE AND MORPHOLOGICAL DETAIL

All episcopic imaging methods produce volume data of high resolution. Except for the fast 3D serial reconstruction technique, all methods permit the detection of organ and tissue details and even cells in the context of whole embryos, parts of embryos, or larger tissue samples. 

Existing episcopic 3D imaging methods use different modalities for enhancing tissue contrasts. Consequently the volume data produced, show dramatic differences in respect to image contrast and data quality. In brief: “Fast 3D serial reconstruction” provides excellent contrasts between bleached tissues and embedding medium. But the images do not permit analysis of tissue architecture. “Epi-3D” permits analysis of anatomical detail and differentiation of tissues, but in general, contrasts are relatively low. Furthermore, tissue contrast between subsequent images frequently differs, because each freshly cut block surface has to be stained individually prior to sectioning. “EFIC” and “SIM_1_” use autofluorescence for detecting cells and tissues. The resulting digital images permit the detection of morphological details and the differentiation of tissues. But contrasts are low and borderlines of anatomical structures are often dubious.

“HREM” and “SIM_2_” use traditional histological dyes for contrasting cells and tissues. Therefore the data quality is high and the appearance of the 2D images is similar to that of the digital images of histological sections, stained with overview dyes. In contrast to histological section series HREM images show delicate shining through artefacts (preceding chapter) and small scratches – the impressions of small irregularities of the edge of the blade. Their reflections appear in all images, because in contrast to histological sections, block faces are not cover-slipped. On the contrary, a series of HREM images is homogenous in respect to tissue contrast, while a series of images captured from subsequent histological sections is inhomogeneous. This inhomogenity is caused by different conditions during processing and staining of the individual sections. SIM_2_ image series also may feature such inhomogeneous tissue contrasts between subsequent images, because each freshly cut block face has to become stained individually prior to image capturing. 

## EPISCOPIC 3D IMAGING METHODS – ANALYZING GENE EXPRESSION PATTERNS

EFIC, SIM_1_, and HREM permit fast analysis of gene expression patterns in the context of morphological detail and tissue architecture [42, 58]. All kinds of samples (embryos, biopsies, tissue samples) can be analysed (http://www.meduniwien.ac.at/3D-Rekonstr/HREM/, http://www.univie.ac.at/efic/). However, for analyzing gene expression patterns the samples must be whole mount stained prior to embedding. The development of whole mount staining protocols is a major challenge. All antibodies and chemicals required must penetrate the entire sample. This is relatively easy with embryos of small size and small tissue samples, but it becomes complicated with larger embryos and bigger tissue samples. 

While EFIC and HREM use a bluish colour reaction, SIM_1_ uses fluorescence antibodies for detecting gene activity. That makes SIM_1 _the only episcopic 3D imaging method that currently permits simultaneous analysis of multiple gene expression patterns in one specimen by using different fluorochromes. However, whole mount fluorescence *in situ* hybridization is still a major challenge. 

Basically also SIM_2_ might be expanded to permit 3D analysis of the patterns of specifically labelled gene products. However, a prerequisite must be the development of protocols and substances for fast on block *in situ* hybridization or immunohistochemical staining. Currently, neither adequate substances, nor protocols do exist.

All episcopic 3D imaging methods that permit the preservation of physical sections, bear the potential to collect the sections and to perform *in situ* hybridization or immunohistochemistry on the sections. After staining, the collected sections can be placed under a microscope and digital images can be captured with a digital camera sitting on the phototubus of the microscope. Proper alignment and equalization of the images of the sections can be achieved by matching them with the corresponding episcopically generated images (e.g. [59]). However, with the exception of the alignment and distortion problems, such an approach has the same limitations as all physical section based 3D reconstruction methods. This is mainly the enormous human resources and the time expense necessary for generating the digital image series. 

## EPISCOPIC 3D IMAGING METHODS – CONCLUSIONS

Episcopic 3D imaging techniques can cheaply and quickly provide precisely registered high-resolution volume data in a highly automated fashion. The data can be used for detailed morphological analysis of biomedical model organisms and for 3D analysis of gene expression patterns in the context of tissue architecture and morphology of embryos, tissue samples and even biopsies. However even the advanced methods are still under construction. The main challenges of the next years will be 1) the development of fully automated, commercially available data generation apparatuses and 2) the modification and standardization of existing, and the development of new whole mount gene expression and gene product labelling protocols. We think that, within the next decades, episcopic 3D imaging will become an indispensable routine tool for researching the genetic regulation of embryo development as well as the genesis of malformations and diseases.

## Figures and Tables

**Table 1.** Characteristics of Modern Episcopic 3D Imaging Techniques. Note that all Specimens have to become fixed and dehydrated prior to embedding

	Fast 3D serial reconstruction (1990)	Epi-3D (1998)	EFIC (2002)	SIM_1_ (2002)	HREM (2006)	SIM_2_ (2008)
**specimens**	bones	embryos, adult material	embryos, adult material, biopsies	embryos, adult material, biopsies	embryos, adult material, biopsies	embryos, adult material, biopsies
**specimen preprocessing**	bleaching	lead acetate immersion during dehydration			eosin staining during dehydration	
**embedding medium**	resin	paraffin mix or resin	paraffin mix	resin	resin	wax or resin
**slice thickness**	≥ 39 µm	≥ ~6 µm	≥ ~1 µm	≥ ~1 µm	≥ ~1 µm	≥0.2 µm (resin) ≥2.5 µm (wax)
**visualization of cells, tissues and organs**	bleaching	lead acetate / sodium-sulfide reaction	auto-fluorescence	auto-fluorescence	eosin	special mixtures for fast staining
**visualization of gene expression**	no	no	yes, if whole mount stained	yes, if whole mount stained	yes, if whole mount stained	currently not
